# Rutin as a Circadian Modulator Preserves Skeletal Muscle Mitochondrial Function and Reduces Oxidative Stress to Protect Against D-Galactose-Induced Aging In Vitro and In Vivo

**DOI:** 10.3390/nu17223571

**Published:** 2025-11-15

**Authors:** Yoonha Choi, Suhyeon Lee, Eunju Kim

**Affiliations:** 1 Department of Nutritional Science and Food Management, Ewha Womans University, Seoul 03760, Republic of Korea; chl9864@naver.com; 2 Department of Food and Nutrition, Kongju National University, 54 Daehak-ro, Yesan 32439, Republic of Korea; suhyeonl22@naver.com

**Keywords:** circadian rhythm, muscle senescence, rutin, oxidative stress, mitochondrial function

## Abstract

**Background:** Skeletal muscle aging is characterized by impaired myogenic differentiation, disrupted circadian rhythms, elevated oxidative stress, and mitochondrial dysfunction. Rutin, a natural flavonoid with antioxidant properties, has been suggested to mitigate aging processes; however, its effects on circadian regulation and muscle homeostasis remain unclear. **Methods:** In vitro, differentiated C2C12 myotubes were treated with D-galactose (D-gal, 20 g/L) with or without rutin (20 μM). In vivo, C57BL/6 mice were supplemented with rutin (100 mg/kg b.w.) via oral gavage in a D-gal-induced aging mouse model (150 mg/kg b.w., i.p.). **Results:** D-gal induced cellular senescence, impaired myogenic differentiation, disrupted circadian oscillations, increased oxidative stress, and compromised mitochondrial function. Rutin treatment restored myotube formation, enhanced circadian rhythmicity of differentiation-related genes, and corrected the antiphase patterns of Per2 and Rorc. It also reduced reactive oxygen species and malondialdehyde levels; increased superoxide dismutase, catalase, and glutathione peroxidase activity; improved ATP production and membrane potential; and decreased mitochondrial oxidative aging, as confirmed by *pMitoTimer* imaging. Furthermore, rutin reinstated the rhythmic expression of oxidative phosphorylation proteins and *Pgc1α*. In vivo, rutin supplementation enhanced muscle performance (prolonged hanging time) and oxidative capacity, particularly at night (ZT14–ZT16), without altering muscle fiber-type distribution, and normalized circadian rhythmicity of core clock genes. **Conclusions:** Rutin attenuates D-gal-induced cellular senescence by modulating circadian rhythms, reducing oxidative stress, and improving mitochondrial function. Importantly, its in vivo effects on muscle performance and circadian regulation suggest that rutin is a promising therapeutic strategy to counteract skeletal muscle aging and sarcopenia.

## 1. Introduction

Skeletal muscle constitutes more than 40% of total body mass and plays an essential role in locomotion, posture maintenance, and whole-body metabolic regulation [1,2,3,4]. With advancing age, skeletal muscle becomes particularly vulnerable to degenerative changes, leading to a progressive loss of mass, strength, and regenerative potential [5,6,7,8]. These changes culminate in sarcopenia, a condition associated with frailty, decreased quality of life, and increased risks of falls, disability, and mortality [9,10,11,12]. Sarcopenia, characterized by progressive loss of skeletal muscle mass and function, is now recognized as a key component of frailty and a predictor of disability and mortality in older adults [13,14]. From a mechanistic perspective, chronic oxidative stress, mitochondrial dysfunction, and redox imbalance play central roles in the pathogenesis of sarcopenia by impairing protein turnover and mitochondrial biogenesis [14,15]. Therefore, targeting redox homeostasis represents a promising strategy for mitigating sarcopenia and age-related muscle decline. Furthermore, age-related muscle decline is driven by impaired mitochondrial protein turnover, accumulation of dysfunctional mitochondria, reduced ATP synthesis, and elevated oxidative stress [16,17,18]. Collectively, these factors compromise bioenergetic homeostasis and exacerbate muscle deterioration [19]. In addition to mitochondrial dysfunction and oxidative imbalance, growing evidence suggests that circadian rhythm disruption is a critical contributor to skeletal muscle aging [20,21,22].

The circadian clock is a fundamental timing system that coordinates physiological processes through transcriptional–translational feedback loops involving core clock genes such as *BMAL1*, *PERs*, *CRYs*, and *RORs* [23,24,25]. It regulates diverse physiological functions and behaviors in humans and animals, with its modulation influenced by factors including diet, physical activity, sleep, endocrine signaling, and metabolism [26,27]. In skeletal muscle, these molecular oscillations regulate mitochondrial oxidative phosphorylation, antioxidant defense, and myogenic differentiation [26,27,28,29,30]. With aging, circadian rhythmicity becomes attenuated, leading to reduced amplitude and stability of gene oscillations and phase shifts that disrupt the temporal regulation of muscle metabolism [31]. This misalignment has been linked to impaired muscle protein turnover, decreased regenerative capacity, and exacerbated oxidative stress, ultimately accelerating muscle atrophy and sarcopenia [31,32,33,34]. Notably, muscle-specific disruption of circadian clock components such as *BMAL1* has been shown to impair the expression of myogenic regulatory factors (*Myf5*, *MyoD*, *myogenin*, and *Mrf4*) and accelerate sarcopenia-like phenotypes, underscoring the direct role of circadian dysregulation in skeletal muscle aging [35]. Experimental models of cellular aging further support this relationship; for example, D-galactose (D-gal)-induced senescence in C2C12 myotubes is accompanied by disrupted circadian oscillations of both core clock and myogenic regulatory genes, diminished myotube formation, and accumulation of oxidative damage [36]. Importantly, the D-gal model not only reproduces oxidative and mitochondrial dysfunction but also recapitulates circadian misalignment, providing a relevant platform for studying circadian–muscle interactions during aging [37,38]. These findings underscore the importance of circadian–mitochondrial homeostasis in maintaining skeletal muscle integrity. Accordingly, restoring age-impaired circadian regulation in skeletal muscle is crucial for preventing and managing muscle atrophy and sarcopenia. Improved circadian alignment reduces oxidative stress and enhances mitochondrial function, thereby protecting muscle health against aging-related deterioration.

Polyphenolic compounds have been extensively studied for their protective roles against age-related dysfunction due to their antioxidant, anti-inflammatory, and anti-aging properties [39]. While certain rutin-containing plant extracts, such as Ginkgo biloba extract (GBE), have shown significant health-promoting effects, including mitigation of chronic neurodegenerative disorders and reduction of extracellular amyloid-β (Aβ) plaques, a hallmark of Alzheimer’s disease [40], the broader therapeutic potential of rutin remains insufficiently characterized in human studies. Rutin, a quercetin glycoside, cannot be absorbed in its native form and is hydrolyzed mainly in the large intestine by caecal microflora [41]. Its oral bioavailability is approximately 20% [42] due to poor aqueous solubility [43]; however, lymphatic absorption has been shown to be more efficient, with slower clearance and prolonged residence time [44]. Structurally, rutin (quercetin-3-O-rutinoside) consists of quercetin linked to the disaccharide rutinose at the 3-hydroxyl position, maintaining potent antioxidant activity through catechol groups on the B-ring [45]. Flavonoids, including rutin, can directly scavenge reactive oxygen species (ROS) by donating electrons and hydrogen atoms to hydroxyl (^•^OH) and superoxide (O_2_^•−^) radicals [46]. In addition, rutin inhibits ROS-producing enzymes, upregulates SOD, CAT, and GPx activities, and reduces lipid peroxidation and oxidative injury [47,48], supporting its potential as a natural antioxidant beneficial against oxidative stress–related muscle aging. In previous studies, rutin has been reported to preserve mitochondrial function in vitro (5–100 μM) and in vivo (25–100 mg/kg) [48,49,50,51,52,53]. However, despite its well-established antioxidant and anti-aging effects, the potential role of rutin in regulating circadian rhythmicity and maintaining skeletal muscle homeostasis during aging remains poorly defined. Emerging evidence suggests that flavonoids may modulate circadian clock function [54,55,56]; however, it is unclear whether rutin can restore circadian rhythmicity in skeletal muscle and thereby influence circadian–mitochondria–oxidative stress interactions. In this study, we aimed to elucidate the relationship between oxidative stress and mitochondrial dysfunction in skeletal muscle and to investigate how circadian regulatory mechanisms modulate these processes.

Despite substantial progress in understanding the molecular underpinnings of muscle aging, significant gaps remain regarding how rutin influences circadian rhythms and mitochondrial function in skeletal muscle. A deeper understanding of this relationship is essential to determine whether rutin can be developed as a therapeutic strategy to target the circadian–oxidative–mitochondrial axis for alleviating skeletal muscle aging and sarcopenia.

## 2. Materials and Methods

### 2.1. C2C12 Cell Culture, Differentiation, and Treatment

C2C12, a mouse myoblast cell line, was cultured in Dulbecco’s modified Eagle’s medium (DMEM) supplemented with 10% fetal bovine serum and 1% penicillin–streptomycin until reaching 90–95% confluence. For differentiation, cells were incubated in differentiation medium (DM) containing 2% horse serum for 4–5 days with 1× Insulin–Transferrin–Selenium (Thermo Fisher Scientific, Waltham, MA, USA) for the initial 2 days. DM was replaced every other day for 4–5 days until full differentiation was achieved. To induce cellular senescence, D-gal (20 g/L; Sigma, St. Louis, MO, USA) was applied, as it causes oxidative stress and has been widely employed to mimic mitochondrial and redox dysfunction in skeletal muscle cells [57,58]. During differentiation, cells were also treated with rutin (20 μM).

### 2.2. Cell Viability Assay

Cell viability was determined using the MTT assay (Thermo Fisher Scientific). Briefly, C2C12 cells (1 × 10^4^/well) were seeded in 96-well plates and treated with rutin (10 or 20 µM) for 96 h. Following treatment, cells were incubated with MTT solution (M2128, Sigma) at 37 °C for 3 h. After removal of the supernatant, the resulting formazan crystals were dissolved in 200 µL of dimethyl sulfoxide, and absorbance was measured at 570 nm using a microplate reader (Tecan, Männedorf, Switzerland).

### 2.3. Hematoxylin and Eosin Staining

C2C12 cells cultured in DM for 5 days were evaluated for myotube formation by hematoxylin and eosin (H&E) staining (Abcam, Cambridge, UK). Following fixation with 4% paraformaldehyde (Sigma-Aldrich, St. Louis, MO, USA), the cells were stained with H&E for cell morphological analysis. Triplicate samples from each group were analyzed histologically, and images were captured using a Leica DMi1 microscope (Wetzlar, Germany). The differentiation ratio was measured using ImageJ software version 1.54.

### 2.4. Immunofluorescence Staining

Differentiated C2C12 myotubes grown on coverslips were rinsed with phosphate-buffered saline (PBS) and fixed in 4% paraformaldehyde in PBS for 10 min at room temperature (RT). After three PBS washes, cells were permeabilized with 0.1% Triton X-100 (GenAll, Seoul, Republic of Korea) in PBS for 10 min and then blocked for 60 min at RT in 5% goat serum. Samples were incubated overnight at 4 °C with skeletal muscle myosin antibody (Santa Cruz Biotechnology, Dallas, TX, USA, sc-32732). Following three PBS washes, cells were incubated for 60 min at RT with goat anti-mouse IgG Alexa Fluor™ 488(Thermo Fisher Scientific, Waltham, MA, USA). Images were acquired using a confocal microscope (Zeiss, Oberkochen, Germany). Negative controls (no-primary antibody and isotype controls) were included to assess nonspecific binding and background.

### 2.5. Senescence-Associated β-Galactosidase (SA-β-Gal) Staining

Senescence in D-gal–treated C2C12 myotubes was evaluated after 5 days of differentiation with a senescence β-galactosidase staining kit (Cell Signaling Technology, Danvers, MA, USA) according to the manufacturer’s instructions. Images were obtained with a Leica DMi1 microscope (Wetzlar, Germany). The proportion of SA-β-gal–positive cells was quantified by counting both total and positively stained cells using ImageJ software (National Institutes of Health, Bethesda, MD, USA).

### 2.6. Intracellular Reactive Oxygen Species (ROS) Detection

Intracellular ROS were assessed using 2′,7′-dichlorodihydrofluorescein diacetate (DCF-DA). Throughout the 5-day differentiation period, C2C12 cells were treated with D-gal and rutin, then incubated with 25 μM DCF-DA for 30 min at 37 °C. Fluorescence intensity was subsequently measured using a Tecan fluorescence microplate reader.

### 2.7. Enzymatic Activities

Intracellular levels of malondialdehyde (MDA) and the enzymatic activities of SOD, CAT, and GPx were analyzed using commercially available kits (Cayman Chemical, Ann Arbor, MI, USA) according to the manufacturer’s instructions.

### 2.8. pMitoTimer Transfection and Imaging

C2C12 myoblasts were transfected with the *pMitoTimer* reporter plasmid (plasmid #52659, Addgene, Watertown, MA, USA) using Lipofectamine according to the manufacturer’s instructions. Following overnight transfection, cells were treated with D-gal and rutin to induce cellular senescence. After 30 h of treatment, mitochondrial images of C2C12 myoblasts were acquired using a confocal microscope (Zeiss, Oberkochen, Germany). The red/green fluorescence ratio, indicative of mitochondrial oxidative status, was quantified using ImageJ software.

### 2.9. MitoTracker Staining

On day 4 of differentiation, C2C12 myotubes were incubated with MitoTracker Red CMXRos (Thermo Fisher Scientific) at a final concentration of 50 nM for 30 min to visualize mitochondria. The cells were then washed twice with PBS, fixed in 4% paraformaldehyde, and mounted with a DAPI-containing solution. Fluorescence images were captured using a confocal microscope (Zeiss, Oberkochen, Germany), and fluorescence intensity was quantified using a Tecan reader.

### 2.10. ATP Measurement

Differentiated C2C12 cells were gently rinsed three times with PBS, and intracellular ATP levels were quantified using an ATP assay kit (Abcam, Cambridge, UK) following the manufacturer’s instructions.

### 2.11. Mitochondrial Membrane Potential (MMP) Measurement

MMP was determined using the cationic dye JC-1 (Thermo Fisher Scientific). In mitochondria with preserved membrane integrity, JC-1 aggregates and emits red fluorescence (Ex/Em = 560/595 nm), whereas depolarized mitochondria retain JC-1 in its monomeric form, emitting green fluorescence (Ex/Em = 485/535 nm). Cells were incubated with 10 μM JC-1 for 20 min at 37 °C in the dark, and fluorescence was measured using a Tecan microplate reader (Männedorf, Switzerland). The MMP value was expressed as the ratio of red to green fluorescence intensity.

### 2.12. Animal Studies

Six-week-old male C57BL/6J mice were purchased from Nara Biotect (Seoul, Republic of Korea). All mice were given a 2-week acclimation period to the environment. The animals were housed in groups of four per cage with free access to food (#D10012g, Raonbio, Yongin, Republic of Korea) and water. Housing conditions included a temperature-controlled room (24 ± 1 °C) and a 12 h light/dark cycle with lights on at 7:00 a.m. Zeitgeber time (ZT) 0 and ZT12 corresponded to light on (7 a.m.) and light off (7 p.m.), respectively. A total of 24 mice were randomly assigned to three groups (*n* = 8/group). Animals in the control group received vehicle treatments, including intraperitoneal (i.p.) injections of PBS and oral gavage of corn oil, to match the administration routes used for the experimental groups. Aging was induced by i.p. injection of D-gal (150 mg/kg b.w.). The selected dosage was based on previous studies using 150–200 mg/kg for 6–12 weeks to induce chronic oxidative stress and aging-like phenotypes [59,60,61]. Rutin (100 mg/kg b.w.) was administered via oral gavage for 12 weeks with D-gal injection. D-gal and rutin were administered during the light phase (ZT0–ZT2) to minimize disruption of the animals’ circadian rhythm. All mice used in this study were included in the analyses without exclusion. Body weight and food intake were recorded weekly. Group allocation was conducted by a technician aware of the assignments. The investigators who performed the experiments were not blinded to group allocation, whereas outcome assessment and data analysis were carried out by independent researchers blinded to the group identities. All animal experiments were conducted in accordance with ethical guidelines for animal care and use and were approved by the Institutional Animal Care and Use Committee of Kongju National University (Approval date: 14 August 2024, Approval No.: KNU_2024-10).

### 2.13. Hanging Test

The hanging test was performed at ZT4–ZT6 and ZT16–ZT18, one week before sacrifice. Mice were placed on a grid, which was then gently inverted over the cage. The latency to fall from the grid was measured. Each mouse underwent two trials, and the mean value was calculated. The maximum test duration was 400 s. To minimize measurement error, standardized measurement criteria were applied.

### 2.14. Muscle Fiber Staining

Muscle fiber-type staining was conducted in the soleus muscle, which has high oxidative capacity due to its rich mitochondrial content. The soleus muscle was selected because it is rich in type I and IIa oxidative fibers with high mitochondrial density, making it particularly suitable for evaluating age-related changes in mitochondrial function and oxidative capacity. Cryosections (12 μm thickness) were cut from frozen calf tissue embedded in optimal cutting temperature and stored at −80 °C until staining. The sections were equilibrated at RT for 10 min and washed in PBS and TBS-Tween for 5 min each. Slides were placed in a staining jar containing hot PBS and incubated inside a Styrofoam box for 5 min. After heating, the slides were left at RT for 30 min and treated with 1.0% Triton X-100 in 1× PBS for 2 min. The slides were then incubated at RT for 1 h in 5% goat serum blocking buffer. During this step, the primary antibody mixture was prepared by combining three antibodies against individual MyHC isoforms (diluted 1:85) (BA-D5, type I; BF-F3, type IIb; SC-71, type IIa) in 1% goat serum primary buffer. The primary antibody solution was applied overnight at 4 °C. On the following day, the slides were washed with PBS and TBS-Tween for 5 min each. The sections were then incubated with the secondary antibody solution (1:200) for 1 h. Coverslips were mounted using fluorescent mounting medium (SouthernBiotech, Birmingham, AL, USA). After mounting, stained slides were observed using a microscope (Olympus, Tokyo, Japan).

### 2.15. Succinate Dehydrogenase (SDH) Staining

SDH staining was performed in the soleus muscle. Cryosections were dried at RT for 10 min and incubated in a solution containing 0.2 M phosphate buffer (pH 7.4), 0.1 M MgCl2 (Sigma-Aldrich), 0.2 M succinic acid (Sigma-Aldrich), and 2.4 mM nitroblue tetrazolium (Sigma-Aldrich) for 10 min at 37 °C. The sections were then washed in deionized water, dehydrated with 50% ethanol, and mounted with Eukitt^®^ quick-hardening mounting medium (Sigma-Aldrich). Stained slides were subsequently observed using a microscope (Olympus, Tokyo, Japan).

### 2.16. Real-Time Polymerase Chain Reaction (PCR) Analysis

In vitro, differentiated C2C12 cells were harvested every 4 h over a 24-h period following dexamethasone (Dex) treatment to monitor circadian oscillations. In vivo, calf muscle collected at ZT6 and ZT18 was pulverized using a tissue pulverizer. Total RNA was extracted using RiboEx RNAsol reagent (GeneAll, Seoul, Republic of Korea), and 2 μg of RNA was reverse-transcribed into cDNA using a cDNA synthesis kit (Thermo Fisher Scientific, Waltham, MA, USA). Gene expression was quantified by real-time PCR using 2× GreenStar qPCR master mix (Bioneer, Daejeon, Republic of Korea) on a Bio-Rad real-time PCR system (Hercules, CA, USA). Relative mRNA levels were calculated using the 2^–ΔΔCt^ method, with GAPDH as the reference gene. Primer sequences are listed in Table 1.

### 2.17. Western Blotting Analysis

In vitro, differentiated C2C12 cells were harvested at circadian time (CT) 6 and CT18 following Dex synchronization to assess diurnal changes. In vivo, calf muscle was collected at ZT6 and ZT18. Cells and powdered calf muscle were washed with cold PBS and lysed in ProtinEx protein extraction buffer (GeneAll, Seoul, Republic of Korea). Protein extracts were separated by SDS–PAGE and transferred to PVDF membranes. Membranes were blocked for 1 h at RT in TBS-T containing 5% skim milk (Bio-Rad, Hercules, CA, USA) and incubated with primary antibodies against ATP5A, MTCO1, and SDHB (Abcam, Cambridge, UK) or GAPDH (Sigma-Aldrich, St. Louis, MO, USA), diluted in TBS-T. After washing, membranes were incubated with HRP-conjugated secondary antibodies, and protein bands were visualized using WestGlow Pico Plus ECL solution (Biomax, Guri, Republic of Korea).

### 2.18. Quantification and Statistical Analysis

Data are expressed as mean ± standard error of the mean (SEM). Statistical analyses were performed using Student’s *t*-test or one-way and two-way ANOVA, followed by Tukey’s post hoc multiple-comparisons test. All analyses were conducted using Prism 10 software (GraphPad, San Diego, CA, USA), and *p* < 0.05 was considered statistically significant.

## 3. Results

### 3.1. Rutin Modulates Core Clock Gene Rhythms Altered by D-Galactose-Induced Cellular Aging in Differentiated C2C12 Cells

**Figure 1:**
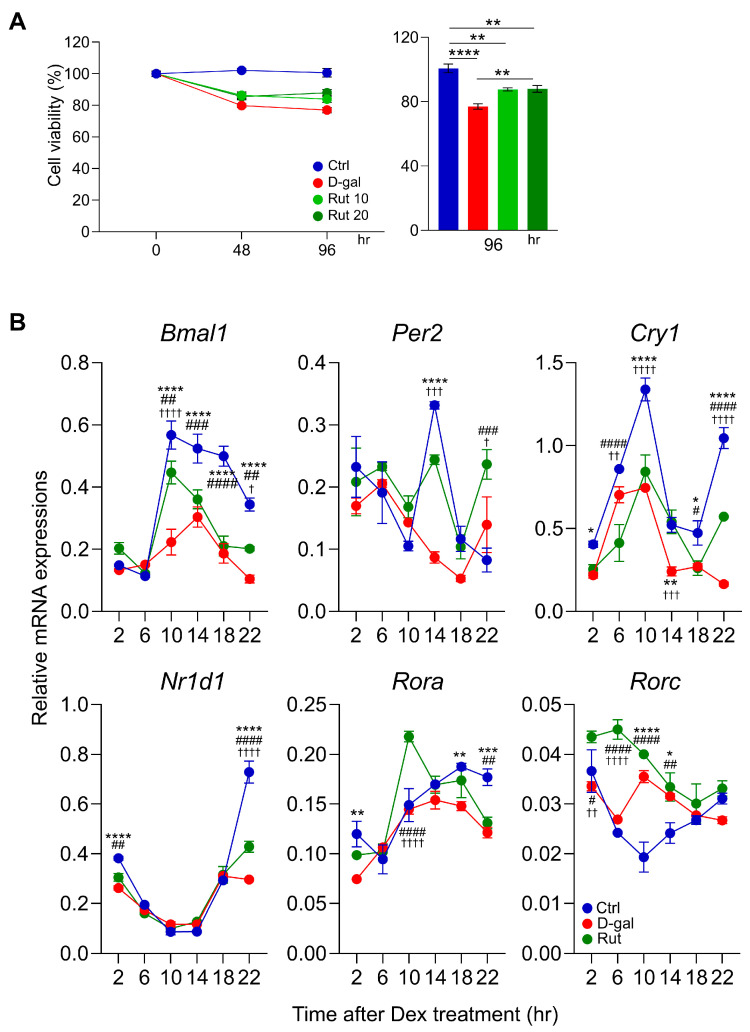
Rutin Regulates Circadian Core Clock Gene Oscillations Impaired by D-galactose-Induced Senescence in Differentiated C2C12 Cells. (**A**) MTT assay was performed using rutin at concentrations of 10 and 20 μM, depending on the experimental time points. One-way ANOVA with Tukey’s multiple comparisons. (**B**) Real-time quantitative PCR was performed to analyze the expression of core clock genes in C2C12 cells four days after differentiation. Data are shown as mean ± SEM every 4 h for 24 h after synchronizing the cells with Dex (*n* = 3/group/time point). *: Crtl vs. D-gal, ^#^: D-gal vs. Rut, †: Ctrl vs. Rut. Two-way ANOVA with Tukey’s multiple comparisons. * *p* < 0.05, ** *p* < 0.01, *** *p* < 0.001, **** *p* < 0.0001, # *p* < 0.05, ## *p* < 0.01, ### *p* < 0.001, #### *p* < 0.0001, † *p* < 0.05, †† *p* < 0.01, ††† *p* < 0.001, †††† *p* < 0.0001.

MTT assays were performed to assess cell viability (Figure 1A). Treatment with D-gal significantly reduced cell viability at both 48 h and 96 h compared with the control group (*p* < 0.0001). In contrast, rutin treatment at concentrations of 10 μM and 20 μM tended to improve cell viability in a dose-dependent manner. Notably, at 96 h, treatment with 20 μM rutin resulted in a significant increase in cell viability compared with the D-gal group (*p* < 0.01). Based on these findings, subsequent experiments were conducted using rutin at 20 μM.

In differentiated C2C12 cells subjected to D-gal–induced senescence, the amplitudes of core clock gene oscillations, including *Bmal1*, *Per2*, *Cry1*, *Nr1d1*, *Rora*, and *Rorc*, were markedly reduced compared with the control group (Figure 1B). Notably, *Per2* and *Rorc* displayed an antiphase oscillatory pattern in the D-gal group relative to controls. Rutin treatment (20 μM) effectively restored these disrupted oscillations. Specifically, rutin markedly enhanced the amplitudes of *Bmal1*, *Nr1d1*, and *Rora*. Moreover, rutin normalized the antiphase pattern of *Per2*, bringing its circadian expression closer to that of the control, and broadly elevated the expression of *Rorc*. These findings demonstrate that rutin exerts a restorative effect on circadian core clock gene oscillations impaired by D-gal–induced senescence, particularly by improving amplitude and correcting phase alterations as a key circadian regulator.

### 3.2. Effect of Rutin on Muscle Cell Differentiation and Circadian Phase Modulation of Muscle Differentiation-Related Genes in D-Gal–Induced Senescence

**Figure 2:**
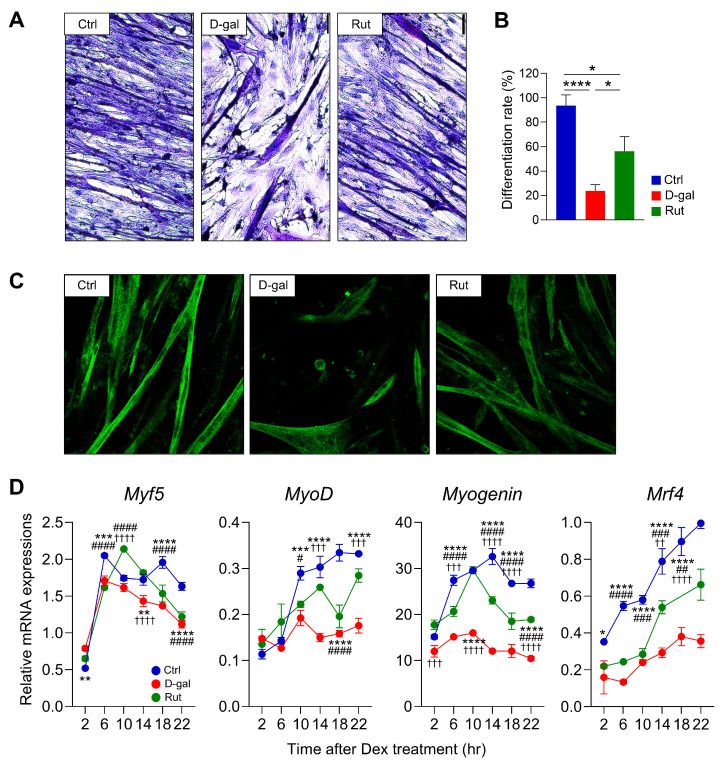
Rutin Promotes Myogenic Differentiation by modulating circadian rhythm in D-galactose-Induced Senescent C2C12 Cells. (**A**) H&E staining at 5 days after differentiation. ×20 magnification. Scale bar = 50 μm. Differentiation ratio (**B**) was measured. Data are shown as mean ± SEM. One-way ANOVA with Tukey’s multiple comparisons. (**C**) Immunofluorescence staining of MCHI. (**D**) Real-time qPCR analysis of genes related to muscle differentiation in C2C12 cells at 4 days after differentiation. Data are shown as mean ± SEM every 4 h for 24 h after synchronizing the cells with Dex (*n* = 3/group/time point). *: Crtl vs. D-gal, ^#^: D-gal vs. Rut, ^†^: Ctrl vs. Rut. Two-way ANOVA with Tukey’s multiple comparisons. * *p* < 0.05, ** *p* < 0.01, *** *p* < 0.001, **** *p* < 0.0001, # *p* < 0.05, ## *p* < 0.01, ### *p* < 0.001, #### *p* < 0.0001, †† *p* < 0.01, ††† *p* < 0.001, †††† *p* < 0.0001.

H&E staining was performed to assess the effect of D-gal–induced senescence on the myogenic differentiation ability of differentiated C2C12 cells after 5 days of differentiation. Myotube formation was significantly decreased in the D-gal group (23.8%) compared to the control (93.6%) (*p* < 0.0001). Rutin treatment (20 μM) partially restored myotube formation, increasing the percentage of myotube-positive cells to 56.3% (*p* < 0.05 vs. D-gal group) (Figure 2A,B). Immunofluorescence staining of skeletal muscle myosin heavy chain further confirmed these results, showing reduced differentiation capacity in the D-gal group compared to the control, while rutin treatment notably increased myosin expression (Figure 2C).

To further assess the molecular basis of myogenic differentiation, the expression of key myogenic regulatory factors, including *Myf5*, *MyoD*, *myogenin*, and *Mrf4*, was analyzed across circadian time points. D-gal–induced senescence markedly suppressed the overall expression levels of these genes and reduced their circadian oscillatory amplitudes compared with the control group (Figure 2D). In particular, rutin treatment restored *Myf5* expression to levels comparable with those in the control group and significantly enhanced the circadian oscillatory amplitudes of *MyoD*, *myogenin*, and *Mrf4*. These results demonstrate that rutin restores and stabilizes the circadian rhythm of muscle differentiation–related genes, thereby counteracting the impaired differentiation potential of senescent muscle cells induced by D-gal.

### 3.3. Rutin Protects Differentiated C2C12 Cells from D-Gal–Induced Senescence via Suppression of Oxidative Stress

**Figure 3:**
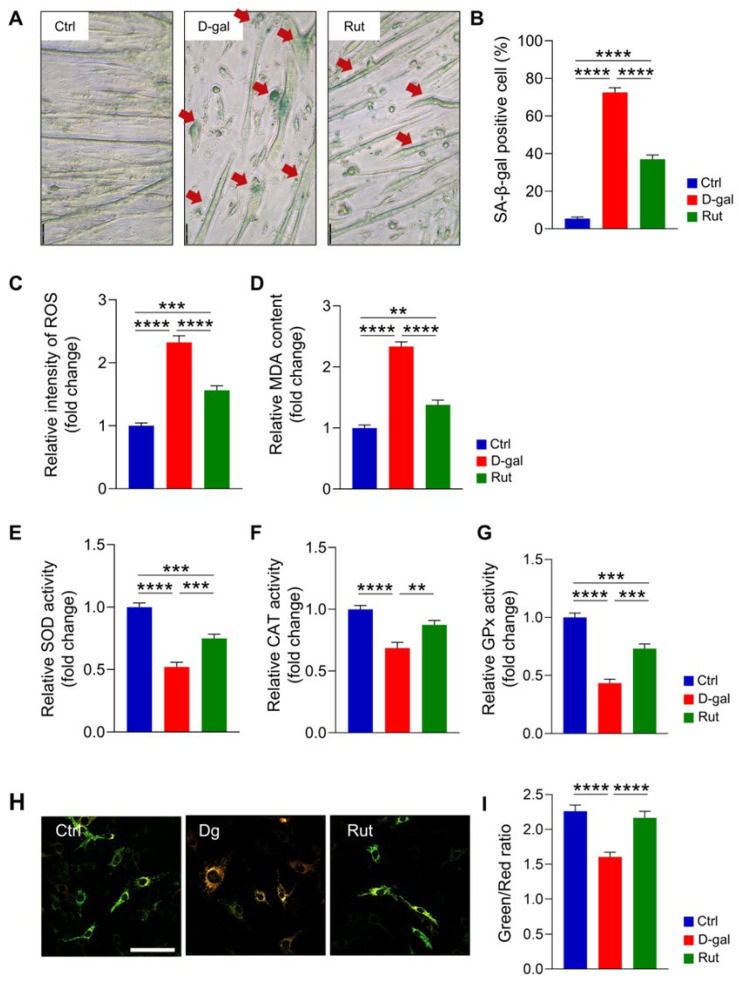
Rutin mitigates D-galactose-induced cellular senescence by alleviating oxidative stress in differentiated C2C12 cells. (**A**) SA-β-galactosidase staining (indicated by red arrows) was performed to assess cellular senescence four days after differentiation. Scale bar = 50 μm (**B**) Quantification of SA-β-gal-positive cells. Data are presented as mean ± SEM and analyzed by one-way ANOVA followed by Tukey’s multiple comparison test. Intracellular ROS levels (**C**) and MDA content (**D**) were also measured (*n* = 6 per group). (**E**–**G**) Enzymatic activities of SOD, CAT, and GPx were determined. Data are expressed as mean ± SEM and analyzed using one-way ANOVA with Tukey’s post hoc test. *n* = 6 per group. (**H**) Representative images of *pMitoTimer* reporter gene expression. Scale bar = 100 μm. (**I**) Quantification of *pMitotimer* Green/red fluorescence intensity. Data are shown as mean ± SEM. One-way ANOVA with Tukey’s multiple comparisons. ** *p* < 0.01, *** *p* < 0.001, **** *p* < 0.0001.

Rutin treatment (20 μM) significantly alleviated D-gal–induced cellular senescence in differentiated C2C12 cells, as demonstrated by SA-β-gal staining (Figure 3A). The proportion of SA-β-gal–positive cells was markedly decreased in the rutin-treated group (37.0%) compared with the D-gal group (72.6%; *p* < 0.0001; Figure 3A,B). Moreover, rutin treatment effectively attenuated oxidative stress markers associated with cellular senescence. Both ROS and MDA levels were dramatically elevated in the D-gal group compared with the control (*p* < 0.0001; Figure 3C,D). In contrast, rutin treatment significantly reduced ROS (*p* < 0.05) and MDA (*p* < 0.0001), highlighting its antioxidant capacity. In addition, the capacities of major antioxidant enzymes, including SOD, CAT, and GPx, were markedly decreased in the D-gal group compared with the control group (*p* < 0.0001 for all; Figure 3E–G). Rutin treatment restored these enzymatic activity levels, significantly improving SOD (*p* < 0.001), CAT (*p* < 0.01), and GPx (*p* < 0.001) activity. To investigate mitochondrial aging, *pMitoTimer* imaging was performed using the reporter construct *pMitoTimer*, which specifically targets mitochondria and fluoresces green when newly synthesized, shifting to red upon oxidation (Figure 3H,I). In D-gal–induced senescent C2C12 myoblasts, the green/red fluorescence ratio was significantly increased compared with the control group, indicating elevated mitochondrial oxidative aging (Figure 3I). In contrast, rutin treatment significantly reduced the green/red ratio, demonstrating its ability to attenuate mitochondrial oxidative aging in senescent muscle cells. Collectively, these findings suggest that rutin protects against D-gal–induced cellular senescence by mitigating oxidative stress and enhancing antioxidant enzyme activity.

### 3.4. Rutin Enhances Mitochondrial Function and Rhythmic OXPHOS Expression in Senescent C2C12 Myotubes

**Figure 4:**
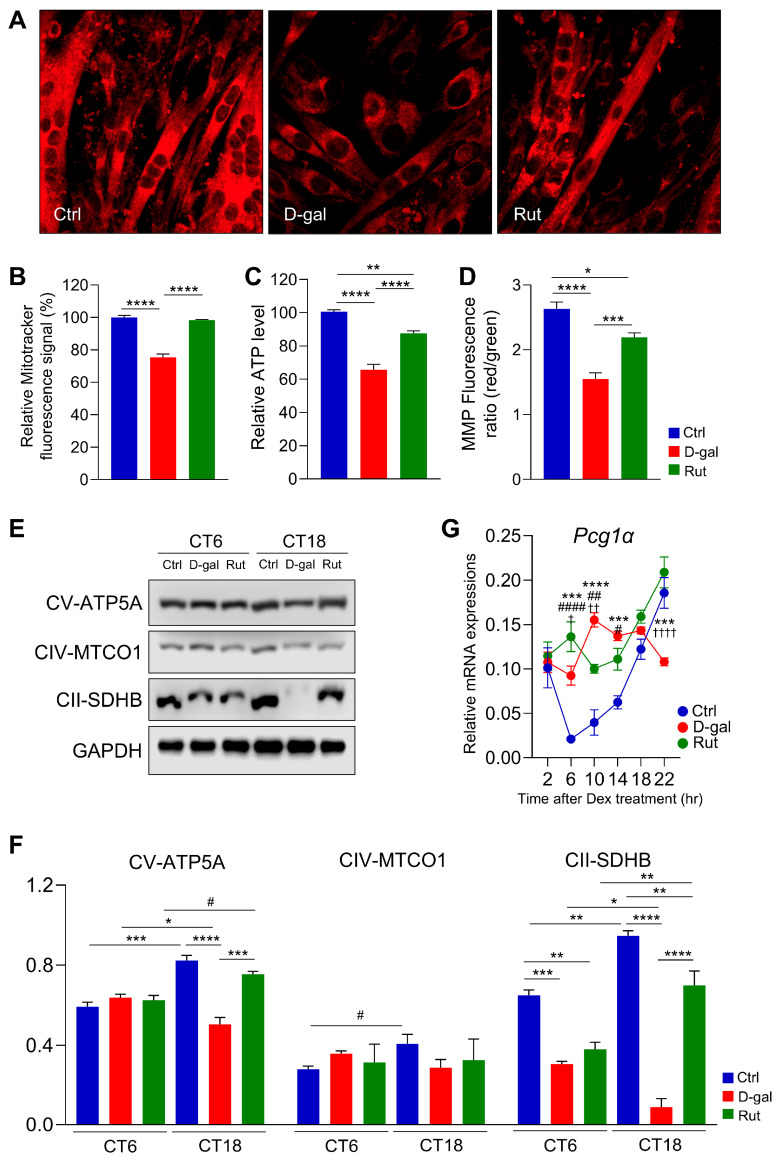
Rutin enhances mitochondrial function in a circadian rhythm–dependent manner. (**A**) Representative confocal microscopy images of MitoTracker staining (×40). (**B**) Quantification of MitoTracker fluorescence intensity. Data are presented as mean ± SEM and analyzed using one-way ANOVA followed by Tukey’s multiple comparison test. Intracellular ATP levels (**C**) and mitochondrial membrane potential (MMP), expressed as the red/green fluorescence ratio (**D**), were determined (*n* = 6 per group). Data are expressed as mean ± SEM and analyzed by one-way ANOVA with Tukey’s post hoc test. (**E**) Representative Western blot images showing oxidative phosphorylation (OXPHOS)-related proteins across diurnal time points. (**F**) Quantitative analysis of OXPHOS protein expression levels. *n* = 3 per group. *: One-way ANOVA with Tukey’s multiple comparisons, ^#^: unpaired *t*-test. (**G**) Real-time qPCR analysis of *Pgc1a* in C2C12 cells at 4 days after differentiation. Data are shown as mean ± SEM every 4 h for 24 h after synchronizing the cells with Dex (*n* = 3/group/time point). *: Crtl vs. D-gal, ^#^: D-gal vs. Rut, ^†^: Ctrl vs. Rut. Two-way ANOVA with Tukey’s multiple comparisons. * *p* < 0.05, ** *p* < 0.01, *** *p* < 0.001, **** *p* < 0.0001, # *p* < 0.05, ## *p* < 0.01, #### *p* < 0.0001, † *p* < 0.05, †† *p* < 0.01, †††† *p* < 0.0001.

To assess mitochondrial content in differentiated C2C12 myotubes, MitoTracker staining was performed. Consistent with the H&E staining results, the control group exhibited well-differentiated myotubes with multinucleated cells and abundant mitochondrial signals (MitoTracker; red; Figure 4A). In contrast, D-gal treatment resulted in poorly differentiated myotubes with markedly diminished mitochondrial staining (Figure 4A). Rutin treatment (20 μM) significantly enhanced mitochondrial content, restoring it to levels comparable with the control group (Figure 4B). Beyond mitochondrial abundance, ATP production capacity was also evaluated. The D-gal group exhibited approximately a 25% reduction in ATP generation relative to the control group (*p* < 0.0001; Figure 4C). Rutin supplementation significantly restored ATP production (*p* < 0.0001 vs. D-gal). Likewise, the mitochondrial membrane potential (MMP), determined by the red/green fluorescence ratio, was markedly reduced in the D-galactose-treated group compared with the control group (*p* < 0.0001; Figure 4D), indicating mitochondrial dysfunction. Treatment with rutin substantially improved MMP, increasing the red/green ratio to levels comparable to the control group (*p* < 0.001 vs. D-gal). Regarding oxidative phosphorylation (OXPHOS) proteins, including ATP5A, MTCO1, and SDHB, the control group displayed distinct diurnal variations, which were diminished by D-gal treatment (Figure 4E,F). In D-gal–induced senescent myotubes, the expression levels of these proteins were notably lower at CT18 than at CT6 (Figure 4E,F). Rutin treatment increased OXPHOS protein expression relative to D-gal treatment, with ATP5A and SDHB regaining a rhythmic oscillatory pattern resembling that of the control group (Figure 4E,F). The mRNA expression of *Pgc1α*, a key regulator of mitochondrial function, exhibited an antiphase pattern in differentiated C2C12 myotubes subjected to D-gal–induced senescence compared with the control group (Figure 4G). Notably, rutin treatment restored the normal oscillatory pattern of Pgc1α expression, indicating its role in rescuing circadian-regulated mitochondrial gene expression in senescent muscle cells. Collectively, these findings indicate that rutin mitigates D-galactose–induced mitochondrial impairment by increasing mitochondrial content, restoring ATP synthesis, enhancing membrane potential, and reestablishing the rhythmic expression of OXPHOS proteins in differentiated C2C12 myotubes.

### 3.5. Effect of Rutin on Skeletal Muscle Function and Circadian Core Clock Gene Oscillations in a D-Gal–Induced Aging Mouse Model

**Figure 5:**
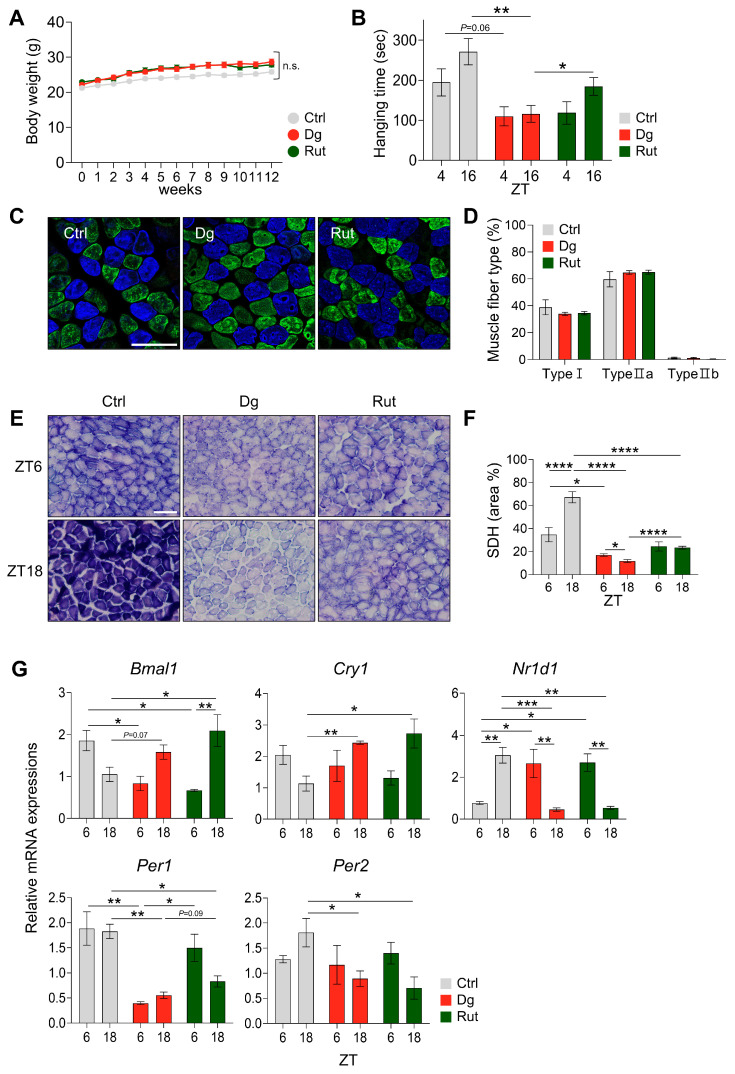
Rutin restores skeletal muscle function and circadian rhythm in D-galactose-Induced aging mouse model. (**A**) Mouse body weight analysis (*n* = 8/group). Data are shown as mean ± SEM. One-way ANOVA with Tukey’s multiple comparisons. (**B**) Hanging time (s) at ZT4 and ZT14 (*n* = 8/group). Data are shown as mean ± SEM. Two-way ANOVA with Tukey’s multiple comparisons. (**C**) Confocal microscopy analysis of muscle fiber type in soleus muscle (Blue: TypeI, green: TypeIIA, red: TypeIIB). 20× magnification. Scale bar: 100 μm. (**D**) Quantification of the proportion of muscle fiber type in soleus muscle. Data are shown as mean ± SEM. One-way ANOVA with Tukey’s multiple comparisons. (**E**) Succinate Dehydrogenase (SDH) staining in soleus muscle. 20× magnification. Scale bar: 100 μm. (**F**) Quantification of the proportion of SDH-positive fibers in soleus muscle at ZT6 and ZT18. Data are shown as mean ± SEM. Two-way ANOVA with Tukey’s multiple comparisons. (**G**) Real-time qPCR analysis of core clock gene expressions in calf tissues at ZT6 and ZT18. Data are shown as mean ± SEM (*n* = 3–4/group/time point). Two-way ANOVA with Tukey’s multiple comparisons. * *p* < 0.05, ** *p* < 0.01, *** *p* < 0.001, **** *p* < 0.0001, n.s not significant.

Weekly measurements of mouse body weight showed no significant differences among groups (Figure 5A). A hanging test was performed to evaluate muscle functional output (Figure 5B). The control group exhibited diurnal variation in muscle performance, with significantly enhanced output at ZT16, corresponding to the active phase. Compared with the control group, D-gal treatment reduced overall motor performance and blunted diurnal variation in muscle function, whereas rutin administration (100 mg/kg b.w.) enhanced motor capacity and prominently re-established diurnal change at ZT16. In terms of muscle diameter, D-gal group showed a trend of decreased diameter than the control group in soleus. Rutin administration significantly increased muscle diameter compared to the D-gal (Supplementary Figure S1A). Muscle fiber staining was performed to analyze histological alterations in muscle fiber type (Figure 5C,D and Supplementary Figure S1B). No significant differences were observed among fiber types in soleus, plantaris, and gastrocnemius. To assess mitochondrial oxidative capacity in D-gal–induced aging skeletal muscle, SDH staining was conducted (Figure 5E). In the control group, mitochondrial oxidative capacity was elevated at ZT18. Conversely, D-gal treatment revealed an antiphase pattern with a significant reduction (*p* < 0.001), which was ameliorated by rutin treatment (Figure 5F). Following these findings, real-time PCR was performed to evaluate core clock gene expression, including *Bmal1*, *Per1*, *Per2*, *Cry1*, and *Nr1d1*, in calf muscle (Figure 5G). D-gal treatment resulted in an antiphase oscillation pattern of *Bmal1*, *Per1*, *Per2*, and *Cry1* compared with the control group. Although the diurnal amplitude of *Bmal1* and *Cry1* appeared higher after rutin treatment, this represents a restoration of rhythmicity rather than abnormal fluctuation, since D-gal treatment markedly dampened their oscillations. Rutin increased *Per1* mRNA levels at ZT6 that were reduced by D-gal treatment by showing a higher diurnal oscillation. In contrast, no significant differences were observed in *Nr1d1* expression between D-gal and rutin groups. Together, these results demonstrate that rutin partially restored and stabilized circadian rhythmicity of core clock genes, improved mitochondrial oxidative capacity, and enhanced muscle functional output, thereby counteracting aging-induced muscle dysfunction.

## 4. Discussion

In this study, we investigated the effects of rutin on the regulation of muscle differentiation, oxidative stress, mitochondrial function, and muscle performance in a circadian-dependent manner in D-gal–induced muscle aging in vitro and in vivo. Our findings demonstrated that rutin supplementation significantly restored circadian oscillations of core clock genes (*Bmal1*, *Per2*, *Cry1*, *Nr1d1*, *Rora*, and *Rorc*) and myogenic regulatory factors (*Myf5*, *MyoD*, *myogenin*, and *Mrf4*), while also improving myotube formation, reducing oxidative stress, and enhancing mitochondrial bioenergetics in aged C2C12 cells. Importantly, these protective effects were also observed in the in vivo D-gal-induced aging mouse model, where rutin partially restored circadian gene expression patterns across time points. In addition, in the in vivo D-gal-induced aging model, rutin ameliorated the disrupted circadian rhythm in skeletal muscle, indicating partial recovery of rhythmicity rather than full restoration. Correspondingly, motor performance, as assessed by the hanging test, was significantly improved, particularly during the mice’s active phase at night (ZT16). These findings indicate that rutin supplementation not only reinstated circadian rhythmicity in aged muscle but also restored diurnal variation in motor performance, which had been impaired by D-gal–induced senescence. Together, these results demonstrate that rutin mitigates D-gal–induced muscle aging by restoring circadian rhythmicity and improving myogenic differentiation, thereby preserving skeletal muscle function.

Circadian rhythms are essential for coordinating energy metabolism, mitochondrial function, and muscle homeostasis [62,63,64]. In particular, skeletal muscle displays one of the strongest peripheral clocks in the body [65,66]. Disruption of these intrinsic oscillations has been shown to impair muscle contractile performance, alter fiber-type composition, and reduce metabolic flexibility [65]. Accumulating evidence indicates that disruptions in circadian regulation contribute to impaired myogenic differentiation, reduced regenerative potential, and sarcopenia [67,68,69,70]. Notably, muscle-specific deletion of *Bmal1* accelerates sarcopenia-like phenotypes by impairing the expression of differentiation factors and altering muscle structure [20,34,71]. Furthermore, aging-associated circadian misalignment has been linked to reduced expression of satellite cell regulators and impaired recovery after injury [35,72]. The interaction between differentiation factors and circadian clock machinery suggests that their dysregulation contributes to the age-related decline of muscle regenerative capacity. In line with these findings, we observed that D-gal treatment disrupted oscillatory expression of both core clock and myogenic differentiation genes, leading to reduced myotube formation and impaired differentiation potential in vitro. Rutin supplementation restored the amplitude and phase alignment of circadian gene oscillations and enhanced the rhythmic expression of muscle differentiation-related genes, thereby improving the differentiation capacity of C2C12 cells. These results suggest that rutin may help stabilize the molecular clock machinery and promote muscle differentiation in aging contexts.

In aging, mitochondrial dysfunction, membrane depolarization, and elevated oxidative stress occur, which collectively reduce muscle endurance and strength and ultimately impair quality of life [73,74]. Numerous studies further indicate that oxidative stress is a key regulator of signaling pathways controlling protein synthesis and degradation in skeletal muscle, thereby contributing to muscle atrophy [75]. Oxidative stress and mitochondrial dysfunction are hallmarks of muscle aging, driving declines in endurance, strength, and overall muscle integrity [74,75,76,77,78]. ROS accumulation during aging promotes mitochondrial DNA mutations, lipid peroxidation, and protein oxidation [79,80,81]. Accordingly, strategies targeting oxidative stress are critical for maintaining muscle performance and mitochondrial function in the elderly. In our study, D-gal treatment markedly increased SA-β-gal staining, a well-established marker of cellular senescence, and was accompanied by elevated levels of ROS and MDA. Concurrently, the activities of key antioxidant enzymes, including SOD, CAT, and GPx, were reduced, thereby recapitulating the oxidative imbalance characteristic of aging. Rutin supplementation effectively reduced oxidative stress, restored antioxidant enzyme activity, and attenuated mitochondrial oxidative aging, as evidenced by *pMitoTimer* imaging. These findings are consistent with previous studies demonstrating rutin’s antioxidant and free radical–scavenging properties [51,82]. Therefore, our results underscore oxidative stress as a central driver of muscle aging and demonstrate that rutin supplementation alleviates this process by restoring antioxidant defenses and reducing mitochondrial oxidative stress. Importantly, this study provides novel evidence that rutin’s protective effects against age-related muscle decline are mediated through dual mechanisms: alleviating oxidative stress both in differentiated muscle cells and within mitochondria.

Mitochondria are particularly vulnerable to oxidative stress, and excessive ROS generation accelerates mitochondrial dysfunction, energy deficiency, and apoptosis in aging skeletal muscle [83]. Mitochondrial dysfunction represents a central mechanism underlying skeletal muscle decline during aging [78,84,85,86]. Age-related alterations in mitochondrial dynamics, including impaired fusion–fission balance and reduced biogenesis, have been implicated in sarcopenia and muscle weakness [87,88,89]. Preserving mitochondrial integrity is therefore crucial for muscle health, as mitochondrial deterioration is a major contributor to sarcopenia [90]. D-gal–induced senescence was associated with reduced mitochondrial content, impaired ATP production, and decreased membrane potential in C2C12 myotubes [36]. Importantly, rutin supplementation restored mitochondrial integrity by increasing mitochondrial content, normalizing ATP production, and improving membrane potential. Moreover, rutin reinstated the diurnal oscillations of OXPHOS proteins (ATP5A, SDHB) and restored *Pgc1α* circadian rhythms, highlighting its role in the circadian regulation of mitochondrial metabolism. Given that *Pgc1α* serves as a master regulator of mitochondrial biogenesis [91] and is under circadian control [92,93,94], its restoration by rutin suggests a critical intervention point in sustaining mitochondrial bioenergetics during aging. In vivo, rutin supplementation improved muscle oxidative capacity, as reflected by succinate dehydrogenase staining, consistent with preserved muscle performance. These results indicate that rutin not only improves mitochondrial efficiency but also restores circadian control over mitochondrial bioenergetics, thereby supporting skeletal muscle function during aging. Our in vivo data further demonstrate that rutin supplementation enhanced muscle performance, as evidenced by prolonged hanging time and increased oxidative capacity, particularly during the active phase (ZT14–ZT16) without change in muscle fiber distribution. Previous studies support this interpretation. For example, Schiaffino et al. (2016) [71] reviewed skeletal muscle–specific *Bmal1* knockout models and reported reductions in muscle mass and metabolic alterations but no quantitative evidence of fiber-type shift. Similarly, Schroder et al. (2013) [95] noted that slow-twitch fibers display stronger circadian rhythmicity of clock-gene expression than fast-twitch fibers, yet direct changes in fiber-type distribution following circadian disruption have not been demonstrated. These findings suggest that circadian misalignment primarily affects metabolic and oxidative functions rather than structural fiber-type transitions, which is consistent with our current results showing no significant change in fiber-type distribution. These effects occurred without alterations in muscle fiber type distribution and were accompanied by partially recovered circadian oscillations and attenuated phase alterations of core clock genes in skeletal muscle.

Collectively, these findings provide an important link between cellular mechanisms and physiological outcomes, indicating that the protective effects of rutin extend beyond cell-intrinsic processes to encompass whole-muscle function. These findings provide an important link between cellular mechanisms and physiological outcomes, indicating that the protective effects of rutin extend beyond cell-intrinsic processes to encompass whole-muscle function. These functional improvements may be attributed to enhanced mitochondrial redox regulation mediated by rutin. Rutin reduced oxidative damage markers (ROS and MDA) while increasing the activities of antioxidant enzymes (SOD and GPx), thereby restoring mitochondrial redox homeostasis. Such redox balance is known to stabilize the molecular clock machinery through the modulation of NAD^+^/NADH-dependent processes and transcriptional regulators such as PGC-1α and RORs, which coordinate energy metabolism and circadian oscillations in skeletal muscle. From a physiological standpoint, these findings highlight that maintaining circadian alignment and redox equilibrium may be critical for mitigating sarcopenia-related muscle decline. Hence, rutin may represent a promising nutraceutical candidate for supporting muscle function in the context of circadian disruption and aging.

However, the precise mechanisms by which rutin restores circadian oscillations remain to be clarified. It is still uncertain whether its protective effects are mediated through direct interactions with transcriptional regulators of the molecular clock or indirectly via mitochondrial- and redox-dependent pathways. Moreover, this study did not address other aging-related processes such as autophagy, inflammation, and proteostasis, all of which are critical for sustaining skeletal muscle health. In vivo, the use of only two time points may be insufficient to capture circadian rhythms in several parameters. Future investigations should therefore explore these additional pathways to gain a more comprehensive understanding of rutin’s mechanisms and to assess its therapeutic potential for preventing and treating sarcopenia through long-term interventions and clinical studies. In addition, given the complex systemic regulation of circadian rhythms and metabolism, further in vivo studies are warranted to delineate the interactions between skeletal muscle and other organs, which may provide additional insights into the integrative inter-organ role of rutin in organismal aging.

Taken together, our study provides novel insights into the role of rutin in regulating circadian rhythmicity, mitochondrial function, and muscle homeostasis during aging. By linking circadian regulation with oxidative stress defense and bioenergetic restoration, rutin emerges as a promising candidate for mitigating skeletal muscle aging and sarcopenia.

## 5. Conclusions

Our study demonstrates that rutin plays a pivotal role in preserving skeletal muscle homeostasis during aging by integrating circadian regulation, oxidative stress defense, and mitochondrial function. Rutin restored circadian oscillations of core clock and differentiation-related genes, reduced oxidative stress, and improved mitochondrial bioenergetics in senescent C2C12 cells. In vivo, rutin enhanced muscle performance and oxidative capacity, particularly during the active phase, while supporting or preserving partial circadian rhythmicity. Collectively, these findings identify rutin as a promising therapeutic candidate for mitigating muscle aging and sarcopenia, warranting further translational and clinical investigation.

## Figures and Tables

**Table 1:** Primer sequences for RT-qPCR.

	Forward (5′–3′)	Reverse (5′–3′)
*Myf5*	CTGTCTGGTCCCAAAGAAC	TGGAGAGAGGGAAGCTGTGT
*Myf5*	CGGCATCTAGAGCCTGGTAG	CTGTCCTCAAAGCTGGGGTA
*MyoD*	AGTGAATGCAACTCCCACAG	ACGATGGACGTAAGGGAGTG
*Myogenin*	GGGCCTCGTGATAACTGCTA	CCTGCTGGGTGAAGAATGTT
*Mrf4*	CCACCTCAGAGCCATTGATACA	GAGCAGGTTTAGTTCCACTTTGTCT
*Bmal1*	CACACTTGCCTCCGAAATAACTC	AGCGCACGGCTGTCTGA
*Per2*	CTGGCGTGGAAGTCATCGT	CTGTCCGCCATTGAGTTCTATG
*Cry1*	GCACCTGACCGAAGACGAAA	GAGCGATCCGCTGACATCA
*Rora*	TCAGCGCCCTGTGTTTTTC	GAGAACCAGGGCCGTGTAG
*Rorc*	CATGGTGCTACTGTGTAAGGTGTGT	CACAGGCGTGCACTCCATAG
*Nr1d1*	CGGCATCTAGAGCCTGGTAG	TGGAGAGAGGGAAGCTGTGT

## Data Availability

The original contributions presented in the study are included in the article/Supplementary Material, further inquiries can be directed to the corresponding author.

## Supplementary Materials

The following supporting information can be downloaded at: https://www.mdpi.com/article/10.3390/nu17223571/s1, Figure S1: Quantification of muscle fiber diameter of soleus and gastrocnemius and muscle fiber type distribution of plantaris.

## Abbreviations

The following abbreviations are used in this manuscript:

CAT	Catalase
CT	Circadian time
DCF-DA	2′,7′-dichlorodihydrofluorescein diacetate
Dex	Dexamethasone
D-gal	D-galactose
DM	Differentiation medium
GPx	Glutathione peroxidase
H&E	Hematoxylin and eosin
MDA	Malondialdehyde
MMP	Mitochondrial membrane potential
PBS	Phosphate-buffered saline
PCR	Polymerase chain reaction
ROS	Reactive oxygen species
RT	Room temperature
SA-β-gal	Senescence-associated β-galactosidase
SDH	Succinate dehydrogenase
SEM	Standard error of the mean
SOD	Superoxide dismutase
ZT	Zeitgeber time
