# Supplementary figures and images for: Rutin as a Circadian Modulator Preserves Skeletal Muscle Mitochondrial Function and Reduces Oxidative Stress to Protect Against D-Galactose-Induced Aging In Vitro and In Vivo
Source: Nutrients. 2025 Nov 15;17(22):3571. doi: 10.3390/nu17223571 (PMC12655827; doi:10.3390/nu17223571)

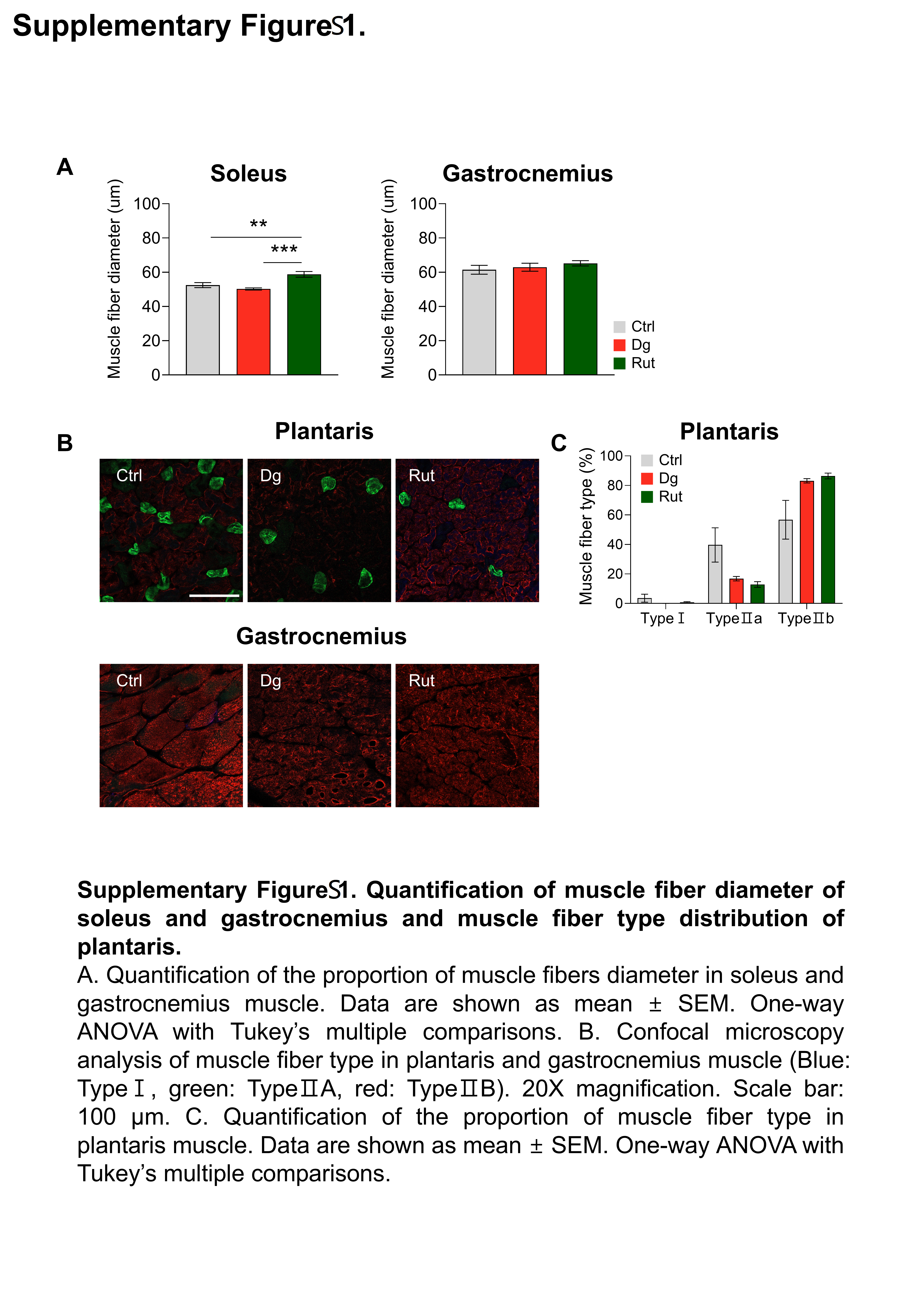

Supplement: Supplementary file 1 [file nutrients-17-03571-s001.zip › nutrients-3929610-supplementary.PNG]
